# The Physiological Requirements of and Nutritional Recommendations for Equestrian Riders

**DOI:** 10.3390/nu15234977

**Published:** 2023-11-30

**Authors:** Russ Best, Jane M. Williams, Jeni Pearce

**Affiliations:** 1 Centre for Sport Science & Human Performance, Waikato Institute of Technology, Te Pūkenga, Hamilton 3200, New Zealand; 2 Department of Animal Science, Hartpury University, Hartpury Gl19 3BE, UK; jane.williams@hartpury.ac.uk; 3 High Performance Sport New Zealand, Auckland 0632, New Zealand; jeni.pearce@hpsnz.co.nz

**Keywords:** equestrian, nutrition, sports nutrition, alcohol, supplements

## Abstract

Equestrian sport is under-researched within the sport science literature, creating a possible knowledge vacuum for athletes and support personnel wishing to train and perform in an evidence-based manner. This review aims to synthesise available evidence from equitation, sport, and veterinary sciences to describe the pertinent rider physiology of equestrian disciplines. Estimates of energy expenditure and the contribution of underpinning energy systems to equestrian performance are used to provide nutrition and hydration recommendations for competition and training in equestrian disciplines. Relative energy deficiency and disordered eating are also considered. The practical challenges of the equestrian environment, including competitive, personal, and professional factors, injury and concussion, and female participation, are discussed to better highlight novelty within equestrian disciplines compared to more commonly studied sports. The evidence and recommendations are supported by example scenarios, and future research directions are outlined.

## 1. Introduction

A recently published bibliometric analysis showed that equestrian sport is underrepresented in sport science literature [1]. Equestrian research tends to focus on rider performance, physiology, and injury rates, with four of the five top-cited equestrian publications at the time of writing focussing on injury prevalence or cases [2,3,4,5], with growing interest in rider position and function [6,7]. For these reasons, the terms equestrian and athlete refer to the human/rider and not equine/horse throughout this review (unless otherwise stated). This bias exists despite recent statistics from the world’s governing body, the Federation Equestre Interationale or International Equestrian Federation (FEI), showing continued global reach and growth in equestrian sport(s) [8]. The lack of academic interest in equestrian performance is further confounded by its being one of the oldest Olympic sports (introduced at the 1896 games), with other disciplines long predating the modern Olympic movement [9,10]. 

Understanding the physiological requirements of equestrian sport via measurement of basic parameters [11] allows nutritionists and sports scientists to calculate pertinent factors, including energy expenditure, quantify the intensity of a session, and provide proportional fuelling recommendations. The majority of data, to date, have been gathered in competitive settings, which clearly differ between disciplines, but may also differ within a rider between different horses and levels of competition [11,12,13]. These data, and therefore nutritional support, may be enhanced by various technologies, e.g., global positioning systems (GPSs), hence their inclusion within this review.

With a rich history, increased participation, and a relative lack of academic investigation, there is clear potential for a knowledge vacuum to form regarding best practices in key areas of equestrian performance [14]. This is compounded by equestrian athletes often prioritise their horses’ welfare and performance over their own [15]. A short-term solution is to borrow and apply knowledge from other sporting disciplines and tailor general recommendations to individual athletes and the demands of their event(s), which will progress in the future to the assessment and development of targeted nutritional interventions for equestrian sport(s). Of further importance is the reported high prevalence of clinical nutrition conditions in equestrian sport, such as disordered eating behaviours and relative energy deficiency in sport (REDS) [16,17,18]. These conditions will acutely impair performance and potentially impart lasting negative physiological and psychological effects [19,20].

Therefore, the aim of the present review is fourfold:

1. Describe the pertinent rider physiology of equestrian disciplines to estimate energy expenditure and the contribution of energy systems to equestrian performance.
2. Provide nutrition and hydration recommendations/guidelines for competition and training for equestrian athletes.
3. Highlight the practical challenges of the equestrian environment.
4. Provide example scenarios to help contextualise points one, two, and three.

## 2. Physiological Requirements of Equestrian Sports

### 2.1. Physiological Demands of Equestrian Sports

There is a small body of literature related to the physiological demands of equestrian sport, enabling some understanding of the energetic requirements across specific disciplines and the variability between disciplines [11,21,22]. Due to data collection constraints, studies may not always account for the realities of equestrian participation, including competing within multiple classes at the same event, and events covering multiple days, which impart further physiological demands. For leisure riders, riding may place expectations on their bodies that they lack sufficient conditioning to tolerate. Riders acknowledge that their fitness affects both their and their horses’ performance [15]. Despite this, few riders report participating in cross-training (or other sports), irrespective of competitive level [15].

Oxygen uptake (V̇O_2_) and peak oxygen uptake (V̇O_2peak_) during equestrian activity are lower than comparable individual or team sports of similar duration, but intensities are sufficient to induce training adaptations that may prove beneficial over time (>60% V̇O_2peak_ [11,23,24]). Rider position on the horse affects energy expenditure, likely because of increased V̇O_2_ through increased muscle mass recruitment; energy expenditure also changes in proportion to gait and jumping requirements [11,22,23,24,25]. Evidentially, when standard equine gaits are combined with complex rider movements, recruiting a larger muscle mass, such as in the cross-country phase of eventing [22] or in Polo [23,26,27], high average (150–185 bpm) and near maximal heart rate (HR) values (~200 bpm) are observed. Yet a dissociation between heart rate and expected V̇O_2_ demand may occur [28,29] during disciplines requiring extended periods in two-point gaits or postures. This is proposed to be due to elevations in blood pressure from repeated isometric contractions, leading to a linear yet disproportionate rise in HR relative to V̇O_2_ requirements [28,30]. This concurrently increases local metabolite flux because muscle metaboreflex stimulation [31] is proportional to muscle activation. Given the localised and non-continuous nature of the isometric contractions experienced during most riding gaits, HR values often exceed %V̇O_2max_ matched endurance activity, but there is likely adequate oxygen supply to facilitate oxidative glycolytic processes and sufficient metabolism of lactate as a fuel source [32,33] during or in recovery from equestrian exercise.

Postural demands of riding have been documented through electro-myography (EMG) and are further supported by measurements including hand grip or core strength [34,35], which may affect rein (tension) or postural control [28,36,37]. EMG data differentiate between levels of rider (elite vs. non-elite; [6,38]) with elite riders demonstrating greater ability to contract musculature actively, independently, and contralaterally [38,39]. This site-specific tonicity likely allows for greater postural control during riding but does not necessarily require greater muscular strength than other athletes or general population controls. Importantly, this is not a rationale to deprioritise strength training in the equestrian schedule; simply, the way strength is expressed as a performance determinant in equestrian disciplines is sport-specific, with benefits to both horse and rider already documented [25,40,41].

Sports science practitioners should aim to decouple the assessment of equine and human physiological loads, seeking to collaborate with equine veterinary practitioners as appropriate. Future research should focus on capturing repeated observations of individual athletes to better understand intra- and inter-athlete variability and how repeated rides, different disciplines, or multi-day competition formats may impact these measures. These factors may then be investigated across the athletic lifespan, either between age groups or by adopting a long-term athlete development model [42,43], as equestrian sports often have youth training and competing against adults [44].

### 2.2. Impact of Environments

Heat exposure leads to an increase in an athlete’s core temperature (Tc) during exercise due to a lowering of the gradient between core and environmental temperatures. Increases in heart rate, sweat rate, and glycogen utilisation occur, ultimately increasing relative oxygen uptake for a given task. Athletes also experience increased thermal sensation, an increased rating of perceived exertion (RPE), and decreased thermal comfort [45,46]. These physiological and perceptual alterations reduce exercise tolerance in proportion to the increase in core temperature [47,48]. Fine motor and psychological skills may also be negatively affected [49,50,51]. Solar load is likely also a factor in equestrian environments and may increase the radiant and reflective heat loads experienced by the athlete during competition [52,53], e.g., in a dressage arena with a light surface.

In addition to environmental heat exposure, equestrian competitive clothing may also incur an additional thermal cost for the athlete. Depending upon the fabrics’ composition and the degree of (required) protection, increased thermal strain due to a reduction in evaporative and convective cooling and an increase in heat storage [54,55] may occur. This remains to be tested experimentally, but studies in tactical athletes or those wearing personal protective equipment demonstrate an increase in perceptual and physiological thermal strain [56,57]. Clothing affects nutritional and hydration requirements in proportion to the insulative qualities of the garments worn, and any additional protective clothing is very important in disciplines where clothing is strictly regulated and shedding layers may be prohibited.

Cold exposure lowers athletes’ Tc and induces vasoconstriction in peripheral regions, increasing blood pressure, heart rate, ventilation (V̇E), and V̇O_2_. Cold impairs performance by worsening exercise economy [58] and increasing cardiovascular strain [59,60], which can be overcome to some extent by habituation [61]. Other impairments are coordination, reaction time, and decision-making [61]; thermal comfort is also reduced [61]. Behavioural strategies (e.g., wearing more layers, extended warm-up time) to attenuate cold are easier and quicker to implement than acclimation or acclimatisation protocols [62] and may be supported by simple nutrition practices, including consuming hot beverages or food pre-event.

The assessment of altitude upon equestrian performance is highly limited, but it is assumed that equestrian performance in events of moderate to long duration and moderate to severe intensity would be impaired at moderate altitude (2000–3000 m above sea level) [63,64], due to the decreased partial pressure of oxygen and decrease in oxygen availability at the working muscle [63]. Of nutritional concern is the potential for nausea, headaches, and diuresis. An athlete’s iron status [64] affects haemoglobin, haematocrit, and ferritin levels, which affect oxygen transportation [64,65]. As with other environmental stressors, responses to altitude are likely to be highly individual, with potential for metabolomic underpinnings [63,66].

### 2.3. Body Composition

There are limited data on body composition in equestrian athletes. Equestrians tend to follow general athletic trends relative to control participants, e.g., greater muscle mass and lower fat mass in the predominant exercising musculature [67,68,69], but equestrians may demonstrate higher fat mass relative to other athlete populations [67].

Rider-to-horse bodyweight ratio is a valid concern given the potential negative impact on horse welfare and performance [70]. However, body mass alone does not capture the morphological characteristics of the rider (e.g., limb and torso length), which would affect saddle fit and further impact a rider’s suitability for a horse [70]. Likewise, a rider’s dynamic stability and balance whilst riding is also an indicator of both performance and their being suitably mounted [6,7,71]. Dyson [70] recommends considering the above in congruence with rider skill level and as part of a multi-disciplinary team to assess the global suitability of the horse-rider dyad. This topic is of particular concern as equestrian sports begin to reflect on their social license to operate and compete [72].

When manipulating body composition, weight-making practices employed in racing are well documented [73,74,75], but are a lesser concern in most other disciplines in the absence of underlying psychological factors. Body composition, combined with rider fitness and postural strength, should consider improving the power-to-weight ratio and minimizing negative impacts on horses’ welfare whilst maintaining athlete health and competitive performance. It is recommended that body composition changes are not solely driven by weight, but form part of a well-rounded strategy incorporating appropriate strength and conditioning practices.

### 2.4. Assistive Technologies

The following section briefly outlines technologies that may assist practitioners in quantifying the internal or external loads experienced by equestrian athletes and how these can inform energy intake or other targeted strategies.

Heart rate (HR) is easily monitored during exercise (via a chest strap or wrist-worn device). Chest-worn devices gather and transmit data via telemetry, usually to a watch [76], whereas wrist-worn devices use optical measurement and reflection to interpret blood flow to derive HR values (photoplethysmography) from light-emitting diodes [76]. As wrist-based measurement is particularly sensitive to movement and may be inappropriate for some disciplines, e.g., eventing or Polo, where chest straps are preferable [26]. HR data are an acute measure of exercise intensity, representing the oxygen demand of exercise due to a linear increase in oxygen consumption up to V̇O_2max_ [77]. Derived measures such as time in modelled HR zones, HR recovery, and HR reserve [78,79] may also assist the athlete or nutritionist in quantifying the cardiovascular demand experienced during training or competition. HR is sensitive to current fitness and training adaptation, environmental conditions, and hydration status and may be influenced by nutritional intervention [48,80,81].

Like HR, portable blood lactate [BLa] measurement allows for the quantification of [BLa] levels and is a proxy for the non-oxidative contribution to exercise performance [33,82]. Given that [BLa] rises proportionally with exercise intensity and increases exponentially beyond V̇O_2max_ [32,33,82], its use within equestrian sport is likely limited to either ‘capping’ exercise intensity, e.g., in an endurance ride, or confirming the energetic nature of disciplines alongside HR and/or respiratory gas measurement. Importantly, whilst respiratory gas measurements provide a quantification of respiratory gas exchange, the use of portable gas analysers in some studies [23] may limit protocol design and reduce external validity. Such detail may feel superfluous in most cases, but is useful when planning energy intake across an extended period, e.g., a tournament, a multi-day event, or when riding multiple horses at one event. When considering both HR and [BLa], practitioners need to be mindful of the metaboreflex and use these measures as guides rather than firm markers in decision-making.

Rating of perceived exertion (RPE) is a measure of an athlete’s perception of exertion, either following task completion or during exercise. There are a range of scales available that have been validated against internal and external [83,84,85,86] measures of exercise performance, such as HR and load lifted, respectively. Given its links to both internal and external load, nutrition practitioners may use RPE as a proxy for global and intra-session fuelling requirements. An important advancement in RPE data collection is that differential RPE values have been assessed acutely and longitudinally [87,88,89], with data derived from athletes’ perceptions of central (e.g., RPE_lungs_), peripheral (e.g., RPE_legs_), and tactical/technical demands. Differential RPE may allow greater precision and individualisation in any nutritional support or education provided given the highly technical nature of some equestrian disciplines, with smaller muscle mass recruitment than team or endurance sports and the compounding effect of riding multiple horses within a day/event.

Global positioning systems have been employed in a range of equestrian disciplines [90,91,92,93] to quantify the external work performed by the horse. The assumption is that the greater the external work performed by the horse (e.g., distance or speed), the greater the energy expenditure of the rider. This has yet to be tested empirically; however, it appears logical, especially when GPS is paired with HR or portable gas exchange measurement (e.g., [11,23,26]). Rider experience/training levels and fitness should be considered in external load assessment, as both contribute to the overall ‘economy’ of riding performance. This encourages equestrian athletes (and practitioners) to move away from a ‘more is better’ perspective of external load and consider skill, task, and discipline-specific demands in greater detail.

Finally, simulators provide the most biomechanically valid method of assessing equestrian activity without the challenges of data collection on live horses [94,95]. Simulator use paired with the methods listed above would likely allow for a thorough estimation of energy expenditure and substrate utilisation across various gaits under controlled conditions and minimise the risk to equipment, equestrians, and horses. This would facilitate the development of normative energy expenditure and substrate utilisation values when riding but comes with a high time and equipment cost, indicating a collaborative approach between athletes and research institutes is needed.

## 3. Nutritional Recommendations for Equestrian Sports

### 3.1. Macronutrient Recommendations

The following sections outline proposed macronutrient intakes for equestrian athletes. Whilst we acknowledge that most athletes and practitioners wish to attain these targets using whole foods, this may not always be achievable, practical, or, in certain circumstances (e.g., deficiency), suboptimal [96]. Athletes are encouraged to consider a food first approach, not a food only approach [96], to sports nutrition in consultation with credible support personnel.

Given the paucity of literature concerning equestrians’ nutrition intake at present, it is premature to comment on specific sports foods and supplements that may align with the physiological demands of equestrian disciplines. Instead, athletes are referred to the Australian Institute of Sport Supplement Framework [97] and the International Olympic Committee and International Association of Athletics Federations consensus statements regarding supplementation [98,99] for existing evidence. Practitioners and researchers are encouraged to conduct feasibility and experimental trials alongside good nutritional practices.

We present recommendations for the total, timing, and type (form) of macronutrient intake and urge readers to consider how food sources can combine multiple macronutrients to conveniently and expediently facilitate performance, recovery, and training adaptation; these are also considered in Example Scenarios (Section 5). The time on a horse should not be a rider’s only nutritional consideration, though, as grooming, care of horses, warm-up, and cool-down practices all confer an energetic cost and need to be accounted for with respect to fuelling and hydration.

### 3.2. Carbohydrate

Carbohydrate intake should be proportional to the competitive and preparatory load being undertaken by the athlete/rider, more recently referred to as ‘fuelling for the work required’ [100]. Daily target intakes are adjusted to suit the logistics of training and competition, as well as factors such as gastrointestinal comfort, food availability, and time until the next session. Table 1 provides some guidelines, with the recommended carbohydrate dose increasing proportional to the number of horses being ridden (volume) and level of work (intensity) a rider performs on a given day. Exceptions to this are where competition or training loads are particularly prolonged, e.g., in endurance riding, three-day eventing, or trekking, as these require additional nutrition preparation and modification of carbohydrate intakes based upon the variation in energy demand for the tasks being performed (e.g., dressage, show jumping, cross-country).

Table 2 provides guidance for carbohydrate feeding for a single competition. Carbohydrate dose (especially fibre) decreases as the competition approaches, then increases post-competition as gastro-intestinal tolerance and appetite typically increase in this window. Nutritional patterns during the competition window may be a response to gut-brain axis communications [101,102], and athlete-specific gut training strategies [103] alongside psychological support may be required in some instances. This is supported by anecdotal reports of riders experiencing discomfort, nausea, and intentionally restricting food prior to competition. Transient carbohydrate exposure in the form of mouth-rinses, gums, gels, and beverages is recommended at competition [104]. These affect the central nervous system, with the brain perceiving increased carbohydrate availability [105,106], and have been shown to increase power production and alertness [107,108,109]. If competition spans multiple hours, regular carbohydrate feeding is recommended, with intake proportional to the duration of competition, provided gastrointestinal comfort is not impaired. Recommended feeding rates (g/h) can be approximated by multiplying the competition duration by 30 g, i.e., 30 g × 2 h = 60 g/h, up to a dose of 90 or 120 g per hour, depending on individual tolerances [103,110,111]. Higher doses should be treated with some scepticism, though, as in endurance sports even lower doses require gastro-intestinal training to be well-tolerated [103,112]. Intra-competition feeding should comprise carbohydrates of multiple monosaccharides e.g., glucose and fructose; this permits greater transportation of carbohydrate across the intestinal membrane, increases exogenous carbohydrate oxidation, and lowers the risk of gastro-intestinal distress [113,114,115,116]. The prevalence and severity of gastro-intestinal distress in equestrians are yet to be examined but are hypothesised to vary between disciplines and competition level due to differences in the proportion of time spent in each equine gait and the need to attenuate and accommodate differing forces depending on event technicality and difficulty. Established gastro-intestinal distress correlates with anxiety and is frequently examined/reported in equestrians [117,118], so it is expected to contribute to both the prevalence and severity of symptoms.

Table 2 also details recommended doses of carbohydrate feeding during recovery from competition, with an initial ~30 g dose seen to improve muscle glycogen resynthesis and attenuate post-exercise immune responses [119,120]. This strategy supports athletes competing in multiple rounds or horses within a day, with a short interval (~1 h) between rides. As recovery continues (≥1 h post-competition), athletes may consume and better tolerate greater carbohydrate doses to support refuelling muscle glycogen stores. Here we recommend consuming 1–2 g/kg of carbohydrate within the three hours following exercise; sooner is preferable. We acknowledge that this relative dose is in contrast to the absolute values provided in other timings listed in Table 2. However, a relative dose allows for greater personalisation and appears more prudent in the absence of large anthropometric datasets regarding equestrian athletes generally and within specific disciplines. Similarly, data are required to better appreciate glycogen utilisation (and resynthesis) in equestrian sports, given variation in discipline demands and the likelihood of competing/riding multiple horses, before more specific recommendations can be made. Ensuring that carbohydrate intake is sufficient post-training and during recovery may contribute to a favourable metabolic environment for anabolism, immune function, and adaptation [121,122,123]; intake should be continued to support refuelling and meet estimated energy requirements. This does not mean a continued high intake every hour during recovery until the next training session or competition. Instead, we recommend transitioning from single-source, small-carbohydrate doses to mixed meals containing larger carbohydrate doses at these intervals and the co-ingestion of carbohydrate and protein to best support continued glycogen resynthesis [119,120]. For some athletes, it may take several hours for feeding to be tolerated. Once such time has passed, normal eating patterns can resume.

This is particularly important during off-season and pre-competition periods where equestrians may be undertaking dedicated off-horse training as well as light to moderate riding work, resulting in an elevated overall training load for the athlete. Similar scenarios may apply for those with grooming and riding jobs alongside their own horse(s).

Finally, athletes should consider the inclusion of sufficient dietary fibre. Recommended intakes vary by national health authority, but typically recommend ~30 g (25 g adult female and 30 g male) of dietary fibre per day [124]. Many athletes do not meet these intakes, despite sufficient energy and carbohydrate consumption [125,126,127], so a focus on fruit, vegetable, and legume intake alongside starchy carbohydrates is warranted. A reduced fibre intake may be useful prior to and during competition if athletes find this improves gastrointestinal comfort [128]. Practically, this should not interfere with meeting carbohydrate recommendations or competition nutrition strategies, but it will mean that some high-fibre foods are limited or avoided and replaced with lower-fibre alternatives. Currently, there are no direct data on the effects of rider position or riding style on the gastrointestinal tract motility or discomfort, but it is hypothesised that there may be similar impacts to those found in distance cycling or high-intensity sprinting due to postural and oscillatory demands [129,130].

### 3.3. Protein

Protein intake facilitates the syntheses of contractile and metabolic proteins, bone, and connective tissues [131,132]. Maintenance of and adaptation to these protein-containing structures requires sufficient dietary intake of essential amino acids, with leucine thought to be a protein synthetic trigger [133,134]. This leads to recommendations of between 1.2 g and 2.2 g/kg/day [132,135], with higher intakes recommended at times of intensified training, reduced energy intake, or injury [135,136] to either develop or preserve muscle mass, respectively. Intake may also be periodised to better support adaptation, as per carbohydrate above. Given the multi-faceted and technical nature of equestrian athletes’ training both on and off the horse, moving beyond categorising an athlete or discipline (e.g., endurance or power focus) is warranted; instead, consider the purpose of the training session and its intended metabolic adaptation(s) and adjust protein intake accordingly. Athletes are discouraged from missing meals and snacks, as this may significantly compromise their intake of protein and fuel.

Specifically, spreading protein intake across the day (four to six doses of 0.25–0.4 g/kg or 25–40 g) appears to be most effective in supporting adaptation, particularly when protein is consumed within 1–3 h post-exercise [137,138]. As muscle remains sensitive to protein intake, ~24 h post-exercise intake can be delayed if not practical. There is some scope to suggest that a larger protein meal prior to sleep (~40 g) may be useful in compensating for a missed meal or optimising overnight post-exercise recovery [139,140]. It is worth noting that for older athletes, muscles may be less sensitive to protein intake, so higher relative and absolute intakes may be beneficial [141,142] and support lifelong exercise and equestrian participation.

The type of protein athletes consume should align with dietary preference and any individual ethical concerns, presuming adequate provision of essential amino acids is achieved. Considered dietary planning is required, particularly in the initial stages of any dietary change (e.g., transitioning to a plant-based, vegetarian, or vegan diet), but is readily achievable under most constraints. Differing protein sources also possess different micronutrient availability, so meals built around a range of protein sources are encouraged. Slightly more plant-based protein sources may be required to be consumed to provide the same impact as animal protein [143]. It is important to acknowledge that much of the research assessing the effects of protein intake post-exercise has used supplements to accurately control the amino acid profile and total amount of protein ingested; the convenience of protein shakes or similar ready-to-drink milk-based products is a useful tool for many athletes, but is not requisite to achieving protein sufficiency.

### 3.4. Fat

Fat is required for healthy cell function and the availability of fat-soluble vitamins. Athletes are recommended to maintain a minimum fat intake of 20% of dietary energy intake; below this, impairment appears to occur [124,144], with potential wider effects upon menstrual and hormonal regulation and omega-3 fatty acid availability [145,146,147]. Similarly, most dietary recommendations suggest a saturated fat intake of ≤10% energy intake [124,144] to minimise risk factors for cardiovascular disease. Dietary fat is commonly reduced when aiming to improve body composition [148]. If restricted consistently, effects are modest for body weight, body fat mass, and waist circumference [149], in proportion to the reduction in fat and energy intake sustained [148]. In athletic populations, fat restriction energy deficits should not occur at the expense of protein intake, as protein will conserve muscle mass even in energy-restricted states [135].

There has been a resurgent interest in high-fat diets in athletic and general populations [150,151]. With the possible exception of endurance riding [29], there does not appear to be a physiologically relevant advantage to consuming a high-fat diet for equestrian athletes. Evidence suggests likely ergolytic or performance-impairing effects [152,153] due to high-fat diets only matching performance at low-moderate endurance intensities and potentially impairing performance at higher intensities relative to sufficient CHO provision [154,155]. This is attributed to decreased enzymatic activity, lowered glycogenolysis, and an increased oxygen cost of exercise for the same output [154,156,157,158]. However, working practices within the equestrian industry [159] and work-riding balance in amateur equestrian athletes [44] may inadvertently increase time spent in ketosis or with low CHO availability through early waking hours and time spent exercising in a fasted state. When ketosis is experimentally induced via exogenous supplementation, aspects of cognitive performance may be impaired [160,161] (we acknowledge that in clinical neurological models, ketosis may exert therapeutic effects [162,163]). As prolonged ketosis in an otherwise healthy equestrian may impair cognitive performance, a ketotic state may increase the risk of workplace or performance-related injury for equestrians through impaired cognition. Testing this hypothesis directly and experimentally is ethically questionable, but more work regarding ketosis and cognitive function in healthy individuals is required before further recommendations are made.

### 3.5. Hydration Recommendations

Euhydration is considered the maintenance of body water, with dehydration being the process of body water loss, leading to hypohydration [144]. Drinking opportunities are episodic, whereas body water losses continually occur due to a range of metabolic processes such as respiration, thermoregulation, and waste excretion [144,164]. To attain euhydration, athletes are recommended to consume 5–10 mL/kg BW two to four hours prior to exercise. This allows sufficient time for the establishment of body water balance or the voiding of excess fluid. During exercise, hypotonic beverages have been shown to maintain body water balance relative to isotonic (*very likely superior*) and hypertonic solutions and water (*likely superior*) [165]. Where beverage intake during/circa exercise is feasible or required, hypotonic fluids are an evidence-based choice.

The beverage hydration index (BHI) was developed to assess the effectiveness of beverages to maintain hydration status relative to water [164]. In situations where long-term maintenance of euhydration is important or there are minimal drinking opportunities, consumption of beverages with a BHI > 1.0 is recommended. Higher BHI values also appear to correlate with the presence of other nutrients and may be particularly indicative of energy content [164], indicating further the practical value of high BHI beverages to athletes and sports nutritionists (as per Table 3).

It is important to acknowledge that some equestrian athletes may commence exercise in a hypohydrated state due to either prolonged riding, hot conditions, or performing multiple rides within a day. Urine specific gravity and/or urine osmolality may be used as spot checks of hydration status, when gathered from mid-stream urine samples (values of <1.020 and <700 mOsmol/kg are considered indicative of euhydration, respectively). Urine colour has been shown to be a reliable and valid measure of hydration status when assessed using the validated chart [166] or recent smartphone applications [167,168]. These are convenient tools for athletes and practitioners looking to improve hydration practices, especially under competitive constraints or high riding volumes.

Heat and solar load negatively impact performance, as reviewed above. Therefore, they are regularly considered during Olympic equestrian events predominantly for horse welfare reasons, with efforts made to minimise exposure to and attenuate high temperatures [169,170]. Due to global television coverage, equestrians may find themselves competing in conditions that are neither conducive to performance nor optimal for horse welfare. Whilst adequate physiological fitness and heat acclimation or acclimatisation strategies will primarily attenuate the deleterious effects of heat exposure [50], either physiologically or perceptually cooling the athlete may afford acute benefits. Pertinent nutrition strategies that mitigate heat symptoms include cold drinks (1.5–5 °C; [171,172]), ice slushies [45,173], and menthol mouth rinsing or ingestion [104,174,175], alongside ensuring adequate hydration. Beverage temperature may only become a consideration when competing in cold or hot environments. Evidence favours the use of cold drinks or ice slushies at temperatures > 28 °C [45]. Menthol mouth rinsing may also be used to perceptually cool athletes in the heat [174,175] but may interfere with thirst sensation over a prolonged period [109]. Solutions are typically between 0.01% and 0.1% menthol and can be co-ingested with ice slushies or as part of a cold beverage [174,176].

### 3.6. Under-Fuelling and Disordered Eating

Given the physiological demands of equestrian sport, the potential for factors that further compound energy expenditure (e.g., riding multiple horses per day, managing life and equine commitments, etc.), and the historical need to conform to a particular aesthetic when competing [177,178], there is potential for equestrians to under-fuel, and develop REDs [18] and disordered eating [16,17]. These factors are best addressed using a multi- and trans-disciplinary approach, given the potential breadth of symptoms and clinical severity if left untreated. However, a general preventative approach, including education regarding energy availability and appropriate and sport-specific body composition assessment for athletes and their support personnel is encouraged [18] prior to any individual health interviews, questionnaire use, or biomarker screening [18]. This approach protects athletes and support personnel, maintains the scope of practice, and encourages appropriate referral.

### 3.7. Micronutrient Recommendations

Although a comprehensive review of individual micronutrients is beyond the scope of the present paper, key micronutrients that have relevance to equestrian athletes and their training patterns are considered. We acknowledge that other micronutrients and supplements may be of interest to equestrian athletes (e.g., [179]), but due to the lack of direct research involving equestrian athletes to date, we have refrained from including them at this time.

#### 3.7.1. Iron

Female athletes have historically been shown to have the highest prevalence of and be at the highest risk of iron deficiency, or anaemia, in athletic populations [180,181,182], with additional requirements of ≤70% compared to general populations [180]. As many equestrian participants are female, this strongly suggests iron is a micronutrient of concern for equestrians and supporting practitioners, with annual monitoring of iron status recommended as a minimum practice. Along with the potential for menstrual iron losses, athletes undertaking large riding volumes may have a lowered energy availability, and post-exercise-inflammatory and/or hepcidin responses may present with varying degrees of iron depletion or post-exercise iron uptake [183,184,185]. Oral contraceptive use does not appear to alter these factors [186]. However, due to horse-riding only being partially weight-bearing and non-osteogenic, these detractors may be less severe than in weight-bearing sports, although this remains to be experimentally tested. Losses in sweat, urine, or faeces may also occur during periods of intensified training or environmental stress. Consuming dietary iron intakes in excess of recommended reference values has been previously recommended (>18 mg for women and >8 mg for men [187]).

Due to the hepcidin response, larger iron intakes, either in meals or via supplementation, are recommended to be timed approximately three to four hours post-exercise, or as is practicable. To further enhance iron uptake, haem iron or non-haem iron paired with vitamin C-containing foods is recommended, with further work required to confirm the place of emerging proteins (e.g., insect and mycoproteins [188,189]) as appropriately bioavailable iron sources for athletic populations.

#### 3.7.2. Vitamin D

Vitamin D, an essential fat-soluble vitamin, influences several aspects of immunity and the regulation of transcription in most tissues [190,191]. Many athletes will obtain ~90% of their vitamin D from UVB exposure from sunlight, which may be seasonally limited by resident latitude and indoor training volume [192], and further limited due to low dietary intake. Given its intracellular role, a deficiency of vitamin D may impart wide-reaching effects. Ensuring adequate vitamin D levels, particularly at times of intensified training or low UVB exposure, is warranted, and blood monitoring of vitamin D status ensures any supplementation can be appropriate and minimises the risk of toxicity [193,194]. Vitamin D status may also improve performance indirectly by reducing the risk and severity of upper respiratory illness, improving muscle function and quality, and working synergistically with calcium to decrease the risk and prevalence of stress fractures [194,195,196].

Generally, supplementation between 800 and 2000 IU/day in winter months if residing above the 35th north and below the 35th south parallel should ensure sufficient vitamin D levels in most individuals [197]. This may be of particular concern for equestrians in the winter months, when training may take place indoors or if training times are limited to early in the morning or late in the evening [198]. Dependent upon the rider’s discipline, coverage by protective clothing worn may also limit sun exposure, potentially providing a further rationale to consider testing levels and supplementation. Those with greater adiposity may also be at risk for insufficiency or deficiency [199,200]. Again, we acknowledge the potential negative impact of larger rider-to-horse ratios upon equine welfare and hope that this risk may be less of a concern with respect to causing reduced vitamin D status in equestrian athletes.

#### 3.7.3. Calcium

Calcium acts across many tissues, serving an array of functions, including bone metabolism, muscle contraction, blood clotting, and nerve conduction. Bone metabolism (growth, repair, and maintenance) is of primary interest in athletic populations, likely due to calcium’s synergistic relationship with vitamin D; combined, they facilitate bone density improvements at doses of 1500 mg/day and 1500–2000 IU, respectively [201]. By extension, reduced calcium intake alongside menstrual dysfunction may increase the risk of fracture and lower bone mineral density resulting from low energy availability in affected athletes [144]. It is not clear that athletes have an additional calcium need compared to non-athletic controls, provided there is dietary sufficiency and energy availability and they are otherwise healthy [98]. Or, more positively, adequate calcium and energy intakes may improve both menstrual and bone health. Dietary and supplement intakes of calcium commonly differ between sexes [202,203,204] and even sporting disciplines [203]—suggesting appropriate education is required to target populations at risk of either deficiency or adverse health outcomes.

These data are particularly important to female equestrians, who may experience various clinical perturbations in calcium status across their participation lifespan, e.g., menstrual dysfunction/osteopenia/menopause. Ensuring adequate calcium and vitamin D intake may decrease the risk of and recovery from fracture if an equestrian were to fall, as evidenced in clinical populations [205,206]. It is unknown whether riding affords a sufficient stimulus to be osteoprotective despite being non-osteogenic due to being partially weight-bearing. This may be addressed by incorporating sufficiently osteogenic off-horse conditioning alongside calcium and vitamin D intake and exposure, but further research is required.

### 3.8. Alcohol and Equestrian Sport

Alcohol consumption within equestrian sport resembles that of the normal population (73%; [15]). Its relevance to equestrian athlete injury is little understood but is documented as a factor within trauma literature [207,208,209], suggesting that alcohol use may impair decision-making and reaction time and increase risk-taking behaviour when riding. Alcohol may also impair body composition due to its caloric value (7 kcal/g) and suppression of lipid oxidation [144,210], promote poor food choices, and further impair thermoregulation and glycogen resynthesis [211,212]. Alcohol may also impair rehydration due to its mild diuretic effects (although this can be manipulated by consuming additional sodium and water concomitantly) [213,214].

Alcohol consumption for coping and pain self-medication has been reported within athletic populations [215]. This is pertinent to equestrian athletes, as in a survey of elite dressage riders (British Group 3 and above), 74% of riders reported competing in pain, with 62% describing this pain as chronic [215]. Likewise, pain was reported in 85% of showjumpers, predominantly in the neck and back, and if athletes competed solely in show jumping their chances of chronic pain more than doubled (odds ratio: 2.2; [216]), indicating a greater likelihood of injury and pain as a result of specialisation and participation in disciplines that involve attenuating higher impact and deceleration forces.

Taking the above into account, alcohol consumption is discouraged pre- and circa- equestrian activity due to its ability to acutely impair performance, mild association with increased risk of injury, and deleterious effects on metabolic processes underpinning successful performance and recovery. Athletes may also wish to abstain from alcohol during intensified training periods and competition to maximise training adaptation and performance, respectively. However, post-competition and as part of a well-chosen diet, moderate alcohol could be consumed after adequate refuelling and rehydration.

## 4. Practical Challenges of the Equestrian Environment

### 4.1. Competitive Challenges

To increase chances of winning or for personal and professional reasons, equestrians often compete multiple horses within or across multiple competitive levels at the same competition. As this reduces the time available for nutritional planning and intake, feeding and hydration strategies need to be easy to implement and well-tolerated. Competitions may also span multiple days (e.g., three-day eventing and multi-day show jumping events), and consideration needs to be paid to nutrition surrounding the competitive window and how general principles can be best adapted to suit the energetic and metabolic demands of each discipline whilst accommodating riders’ nutritional and taste preferences. We strongly encourage support practitioners to attend competitions to see how individual athletes manage these constraints, allowing for better tailoring of support to individual athlete circumstances and routines. However, given the amount of (inter)national travel in mid- to high-level equestrian sport, remote support delivered through social media or messaging apps should be considered. Establishing a connection with riders is essential for support practitioners [217,218,219].

Additional research is required to understand the periodisation structures employed across seasons, within and between equestrian disciplines, and how this is reflected in the nutritional needs of the human athlete. As with other sports, there is likely to be an inverse relationship between training volume and training intensity; however, this will be multiplied by the number of horses ridden and any off-horse/non-riding conditioning that provides an added energetic demand. The guidance presented above regarding macronutrient consumption, particularly carbohydrate availability, should be employed before consideration of possible/potential supplementation strategies in accordance with the physiological demands of the event and training phase. Athletes are reminded that many equestrian disciplines require compliance with the World Anti-Doping Agency code, and there is a near absence of supplementation research in equestrian settings to date.

### 4.2. Personal and Professional Challenges

Unlike other professional and semi-professional athletes, equestrian athletes have a tangible need to earn a living to sustain themselves and to provide sufficiently for their horses’ needs [220,221]. This pressure needs to be respected as it directly influences available income for purchasing food (and specialist sports nutrition products) and engaging with nutrition professionals, unless supported by a sponsor(s) or regional or national governing body. These constraints must be at the forefront of the nutritionist’s mind, but they do not prevent good nutrition practices from being implemented. Athletes may also train and/or breed horses as a source of income, potentially increasing their energy expenditure beyond that of their training and competitive load. Further constraints may include travel to and from stabling facilities, irregular or unfavourable working hours, and time pressures that lead to the prioritisation of care for equine over human athletes [44,220,221].

### 4.3. Injury and Concussion

Injuries whilst riding or working with horses are relatively common and range from mild to severe in nature, with falling, being kicked, or a head injury being the most commonly reported injuries [208,222,223]. Concussions are an obvious concern, with an incidence documented at 0.19 per 1000 h of riding [224]. Following injury, there is an acute increase in energy expenditure due to the metabolic demands of healing (including inflammation, proliferation, and remodelling [136]). If the injury is sufficiently severe, the athlete will likely experience a period of immobilisation or bed rest, which often leads to losses of muscle mass [136]. Energy expenditure when immobile is reduced if the injury is prolonged enough to warrant lengthy bed rest; thus, a concomitant reduction in energy intake may be needed. Although in the initial recovery stages, energy expenditure is likely elevated by 15–50%, proportional to the degree of trauma sustained [136,225]. In the recovery period, where athletes may transition from being immobile to using mobility aids (crutches, etc.), the energy cost of ambulation will increase by two- to three-fold [226], in comparison to healthy locomotion. Energy needs during recovery should therefore be matched to both the time since injury, injury severity, and degree of locomotion/immobilisation experienced by the athlete, with an emphasis on protein intake and distribution to support muscle retention [136,225]. Regular review and modification are advised. During recovery from injury, there may also be scope for targeted supplementation to support connective tissue repair, alongside physiotherapy or similar rehabilitation, in the form of gelatine and vitamin C supplementation [227]. Gelatine is a source of the amino acids proline and glycine, which form collagen, a constituent of connective tissue, with vitamin C acting as a co-factor to increase collagen synthesis via hydroxylation and gene transcription [228]. Supplementation has been shown to augment collagen synthesis and tendon function following training and/or injury [227,229] at a dose of 15 g of gelatine and 48 mg of vitamin C.

Concussion is a concern in equestrian sports, with recent publications highlighting the prevalence and mechanism of concussive injury [224,230]. Concussion in equestrians (0.19 concussions per 1000 h [224]) is approximately half that of soccer match play (0.44 concussions per 1000 h [231]), but may be more severe given that impact, rotational velocity, and linear and rotational acceleration are considered significant predictors of equestrian concussion [230]. These parameters are likely greater than those experienced by soccer players, at least during riding, especially when considering most riders sustain concussions whilst wearing a helmet [224].

Omega-3 intake through fish or krill oil supplementation appears to be an effective complementary nutritional strategy with respect to concussion at present [232], with improvements in axonal and neural damage, oxidative stress, and functional outcomes being noted [232,233]. Creatine is another supplement with possible benefits based on recent reviews [234,235]. Given the risk of concussion in equestrian athletes and the potential systemic benefits of consuming sufficient omega-3 [236], considering general or prophylactic supplementation in conjunction with dietary sources such as oily fish and high-fat plant foods and, to a lesser extent, grass-fed animal proteins (meat, eggs, etc.) and soybeans may be worthwhile. Omega-3 supplementation presents a likely small health benefit and low risk of harm to the athlete in the absence of contraindications, with potential for positive effects if concussion is sustained. However, given the complexity of concussion diagnoses and the likelihood of accompanying injury in equestrian athletes, seeking appropriate clinical and rehabilitation advice prior to pursuing dietetic support if a concussion is sustained is recommended. Readers are referred to the UK concussion guidelines for sport [237].

### 4.4. Female Participation

Recent data from the UK and USA suggest that ~95% of regular equestrian participants are female [15,44]. This is a possible explanation for the general lack of academic interest in equestrian disciplines, given the now well-documented gender bias in sports medicine and science research [238]. The volume of female equestrian participants presents a valuable opportunity to better understand female athletes, which should be celebrated and capitalised upon.

There is a body of work pertinent to the female equestrian in breast pain, breast biomechanics, and consequent bra recommendations [239,240]. This work notes that ~40% of female equestrians experience breast pain, with 21% reporting negative effects on performance [240]. Pain experienced is linearly related to self-reported cup size [239,240]. However, when proper breast support is provided larger cup-sized athletes see the greatest improvement in biomechanical parameters [239].

Menstrual cycle phases are thought to impact exercise performance via alterations in female athletes’ hormonal profiles, affecting systemic physiology [241]. It is reasonable that this may extend to factors affecting equestrian performance. Physiological and cognitive performance have been shown to vary, sometimes considerably, between menstrual cycle phases [242]. The hormonally driven change in body temperature between menstrual cycle phases may also have an impact on hydration needs and comfort. Given the highly individualised nature of the menstrual cycle, much more research is needed. Recommendations to modify nutritional intake by menstrual cycle phase at present are contentious, provided energy, CHO, and protein needs are sufficient [243]. An athlete’s energy and macronutrient and fluid needs provide a strong platform for the sports nutrition practitioner to individualise or modify nutritional support for the (female) equestrian athlete, acknowledging that athletes will have variations in their experience of menstrual symptoms [242].

## 5. Example Scenarios

The following scenarios are provided as exemplars of how the above nutritional guidelines may be modified across single- or multi-day competitions with varying physiological demands. These are a guide and should be individualised alongside appropriate professional support and trial and reflection in training. To contextualise the likely energy requirements of events, Table 4 provides published energy expenditure ranges for riders when riding across equine gaits. We acknowledge that endurance riding is not listed here due to its unique and prolonged competition format. Williams’ recent review provides an appropriate resource to understand and assess individual strengths, weaknesses, and opportunities for improvement [29].

### 5.1. Show Jumping

**Figure 1:**
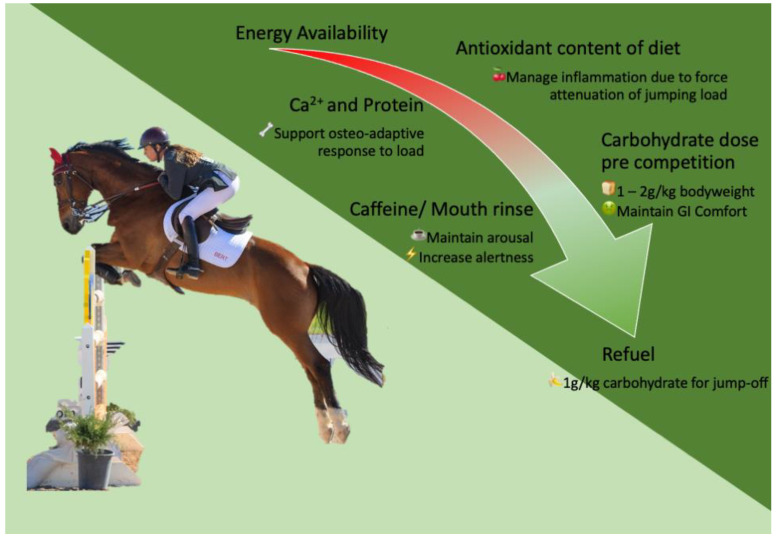
Nutritional strategies to support show jumping.

Show jumping is typically performed as a single, short-duration ride that, like dressage, may be considered highly technical. Performance class is determined by the height of the jumps presented and the technicality of the course, with performance comprised of a combination of time to complete the course and any performance faults (jumps down or refusals). Due to the lower event duration (<90 s), show jumping will have a lower total energetic demand than some other disciplines [22], but stands to benefit from nutrition strategies that maintain alertness and arousal [117]. Most recreational competition formats will employ a heat and final on the same day, and riders may jump in multiple classes, so the need for post-ride fuelling is still apparent. Show jumpers have an exaggerated need to attenuate force through take-off and landing [215] compared to other disciplines, suggesting adequate energy availability, anti-oxidant content, and sufficient calcium and protein for osteogenesis are requisites within the training diet of show jumpers. These recommendations are detailed in Figure 1.

### 5.2. Polo

**Figure 2:**
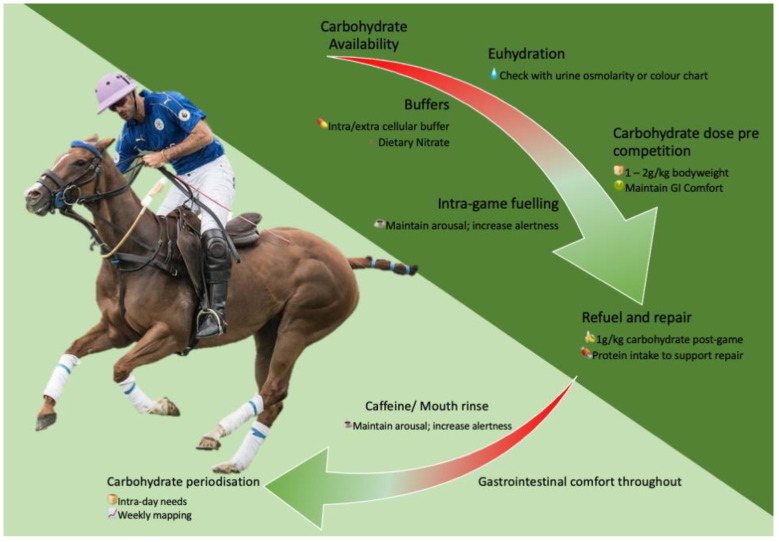
Nutritional strategies to support polo.

Polo play consists of relatively long (30–60 min) games, separated into seven-minute chukkas, characterised by high speeds, accelerations, and decelerations [90,248]. Rules dictate multiple horse-rider pairings are required [221], and tournaments often take place over weekends at recreational levels, or professionals may play multiple tournaments concomitantly to have a large weekly playing volume [26,249]. HR data confirm the high intensity of play [26,27], indicating a potential use for buffering supplementation or dietary nitrate [250,251,252]. This also suggests a predominantly carbohydrate-based fuelling strategy, periodised to reflect games played within a week, and an intake relatively higher than other disciplines. Adequate protein intake post-match is required due to the physical contact that takes place within games. The separation of polo matches into chukkas presents an opportunity for intra-game fuelling, supported by oral sensing strategies to maximise perception of readiness whilst minimising the risk of GI disturbance [104]. This can be extended with inter-game fuelling for professionals playing more than one game per day. Ensuring euhydration prior to games commencing is also paramount due to the extended game time [253] and the potential resultant heat gain to players via contact with horses acting as a large heat conductor due to the mechanical load experienced when performing accelerations and decelerations and running at high speeds [254,255,256]. These strategies are summarised in Figure 2, below.

### 5.3. Eventing

Eventing is perhaps the most logistically and metabolically complex equestrian discipline, comprising three distinct disciplines/phases and taking place in either single, two, or three-day formats [13,257]. This emphasises the need for personalisation and the convenience of any suggested nutritional strategies for riders. Each phase (dressage, show jumping, and cross-country) possesses distinct metabolic demands, which increase linearly in proportion to performance velocity and corresponding cardiovascular intensity (e.g., [La] levels and V̇O_2_ [22]), suggesting practitioners increase their consideration of recovery and relative energy intake between phases and across each day compared to single-effort disciplines. As with other single-horse disciplines, there is also the potential for riders to compete in multiple classes, further compounding energetic requirements, especially if fuelling opportunities are limited. Those supporting eventers, while considering the strategies outlined above, should adopt a nutrition periodisation timeline more akin to team sports practitioners, using competition day(s) as Day 0 and prioritising carbohydrate availability in the days prior to competition, i.e., event day 2, event day 1 [258], with in-competition nutritional strategies aligned with phase metabolic and skill demands and duration and prioritising recovery in accordance with the 4 R’s model (Rehydrate, Refuel, Repair, Rest [259]). This aims to maximise hydration and retain muscle glycogen levels prior to, during, and following performance (as per [260]), regardless of whether a single or multi-day format is adopted.

## 6. Future Directions

Several gaps exist within our current understanding of the equestrian athlete and present opportunities for future research and enhanced support at governing body levels, with a view to providing discipline-specific recommendations.

1. In order to maximise the efficacy of the nutritional recommendations above, we need to assess the gastrointestinal symptoms experienced by equestrian athletes and develop gut training strategies at an individual level.
2. Studies are required to assess the effects of nutritional interventions to support equestrian athletes’ performance and wellbeing in training and competition.
  - This may also comprise a wider dietary and physiological assessment.
  - Commonly used, evidence-based supplements matching assessed or perceived physiological event demands may present an appropriate starting point for interventions.
3. Given the relative paucity of evidence for best practices, an integrated approach between performance personnel supporting equestrian athletes is required. Some challenges faced by equestrian athletes are best addressed by a range of practitioners, as opposed to a single point of ownership. Having the professional and intellectual humility to accept and encourage this will hopefully lead to better outcomes for all stakeholders.

We should seek to optimise the performance and training of human athletes, not just for their own sake, but as poor nutrition and hydration impair decision-making, the potential for indirect improvements in horse welfare exists. This further extends equestrian sports social license to operate beyond the horse and rider dyad into the wider employment of the equestrian industry (e.g., work riders, stable staff), who are often overlooked but would benefit from improved nutrition and hydration practices.

## 7. Conclusions

The nutrition requirements of equestrians are likely not dissimilar to those of other athletes, but further understanding of equestrian physiology and the training environment is required to make more precise recommendations. Macronutrient intake should be modified in accordance with competition and training load. Hydration may be an underappreciated factor in equestrian sports, with further research required in this area. Moderate alcohol consumption can be enjoyed, provided it does not increase the risk of injury to the athlete or horse(s). Practical fuelling strategies integrated with lifestyles are critical. Nutrition practitioners working with equestrian athletes should consider not only the underpinning physiological requirements but also the unique horse-rider relationship(s) required to produce successful equestrian performance whilst being mindful of the practical challenges that accompany equestrian participants’ disciplines.

## Figures and Tables

**Table 1:** Daily carbohydrate recommendations for equestrian athletes.

Riding Volume	Intake (g/kg/day)	Scenario
*Low*	3–5	Recreational riding or low-energy-related tasks, e.g., lunging. Active recovery or off-horse training days
*Moderate*	5–7	One or more horses in light- to medium-ridden work. Flat work and short-duration technical sessions.
*High*	7–10	Multiple horses in medium-to-heavy work. Higher skill and discipline-specific sessions e.g., stick and ball or show jumping.
*Very High*	≥10	Multiple horses in heavy work and/or full competitive load, likely competing across multiple classes, days, or extended periods, e.g., endurance, eventing.
*Additional Context*	Where higher intakes are required, multiple forms and sources of carbohydrate are likely optimal and better tolerated.	Gut training is recommended in training to assess and establish tolerance for intake circa and during competition.

**Table 2:** Acute carbohydrate feeding rates and strategies.

Timing	Pre-Event (−1 to −4 h)	Circa-Event	Between Rounds/Immediately Post (≤1 h)	Post-Competition (≥1 h) ^1^
*Dose*	30–60 g/h	Mouth rinse/<30 g	30 g–60 g/h	1–2 g/kg
*Event example*	Arrival at ground; horse unboxed and yarded	Post-human warm-up prior to mounting	As per timing and as required and tolerated	Close to competition preferred; combine with hydration and protein intake; increase consumption as tolerated
*Rationale*	Adequate fuelling for the event demands GI comfort Practical timing alongside horse management	Stimulates oral CHO receptors and associated brain regions	Increased exogenous CHO oxidation, partial sparing effect	Increased rate of glycogen resynthesis; convenient co-ingestion with other recovery fundamentals

^1^ This denotes post-competition when rounds of competition are separated by >1 h and may enhance the opportunity for acute glycogen resynthesis. This may extend to a period that is practical and best positions the athlete(s) to maximise glycogen stores whilst maintaining a realistic energy intake, alongside other recovery strategies.

**Table 3:** Beverage hydration index, energy, carbohydrate (CHO), protein (PRO), and fat content of commonly consumed beverages; adapted from [164].

Beverage	BHI	Energy (kcal/100 mL)	CHO (g/100 mL)	PRO (g/100 mL)	FAT (g/100 mL)
Water	1.0	0	0	0	0
Cola	1.4	42	10.6	0	0
Sports Drink	1.0	16	3.9	0	0
ORS *	1.5	8	1.8	0	0.1
Coffee	0.9	0.4	0.1	0	0
Full fat milk	1.4	64	4.7	3.2	3.6
Skimmed milk	1.5	35	5.0	3.4	0.1

* ORS: oral rehydration solution.

**Table 4:** Rider energy expenditure for typical equine gaits; speeds are sourced from [244,245,246]; energy expenditure values from [11,247].

			Energy Expenditure (kcal/h)		
Gait	Typical Speeds (km/h)	Energy Expenditure (METS)	60 kg Athlete	70 kg Athlete	80 kg Athlete
Walk	7	3.4	204	238	272
Trot	13	6.2	372	434	496
Canter	16–27	7.7	462	539	616
Suspended Canter ^1^	as above	10.65	639	746	852
Gallop	≥40	9.4	564	658	752
Show jumping	N/A	11.04	662	773	883

^1^ Additional energy expenditure during suspended canter is a result of the rider having to propel and control their own bodyweight vertically in unison with the horse’s gait, as opposed to sitting to the canter, which only requires accommodating the horse’s motion.

## Data Availability

No new data were created or analysed in this study. Data sharing is not applicable to this article.
